# Effect of distance education courses held in different environments on emotions of the instructor

**DOI:** 10.1371/journal.pone.0295935

**Published:** 2024-01-26

**Authors:** Güray Tonguç

**Affiliations:** Applied Sciences Faculty, Information Management Systems Department, Akdeniz University, Antalya, Turkey; Golestan University, ISLAMIC REPUBLIC OF IRAN

## Abstract

In this study, the emotional states of the instructors who teach by distance education and the effect of the environment in which they give the lesson on their emotions were examined. Computer-aided "Facial Action Coding System" method was used to measure emotion values from facial images. Through the software developed by the researchers using the Microsoft Face Recognition API, 43292 facial images taken from five trainers during their training were analysed and seven basic emotions representing facial expressions were obtained numerically. As a result of the analysis, it was found that the emotions of the instructors that can be described as negative in the lessons held in the e-studio environment generally increased at the beginning of the lesson, decreased in the following minutes and increased again at the end of the lesson; On the other hand, it was determined that positive emotions decreased at the beginning of the lesson and increased later. In the home environment, while the emotions that can be described as negative at the beginning decreased, positive emotions increased. A significant difference was determined between home and e-studio environment in all emotions except anger. One of the emotions with a difference in value between the two environments is happiness, and it has been determined that happiness has higher values in the home environment. It has been determined that other emotions are experienced more in the e-studio environment. It is thought that the results of the study will contribute to the mental states of the instructors who teach through distance education and to the efficiency of distance education.

## Introduction

Emotions are regarded as a motivational energy source in individuals [1]. Emotions create different cognitive, affective and behavioural changes in individuals [2]. The effect of emotions on education and learning processes cannot be ignored. Education and learning processes are an important part of emotions and are in a mutual relationship that affects each other. Emotions affect students’ interest in learning, motivation and level of participation [3]. A learning environment in which students experience positive emotions enriches their learning experiences and leads to deeper learning [4,5]. Stress or negative emotions experienced during learning can distract students and negatively affect learning [6]. Teachers’ approach to students with sincere interest and compassion ensures a safe learning environment. In this environment, students experience better learning and become more willing to learn. In addition, positive teacher emotions can increase students’ self-confidence and positively affect their academic success [7,8]. On the other hand, negative emotional states can negatively affect teachers’ reactions to students and classroom management [9].

Teachers go through a variety of emotional experiences in the classroom. Emotions such as professional satisfaction, pride in student achievements, anxiety in the face of students’ difficulties, and sometimes fatigue and disappointment can take place in teachers’ daily experiences. It is known that teacher emotions are related to education and learning processes, and that teachers’ emotional experiences have a significant impact on students and classroom environment [10]. Teachers’ emotional state can affect interactions in the classroom and relationships with students, thus having an indirect impact on education quality and student achievement [11–13].

### Previous works

Emotions are very important for students and teachers, who are two important components of learning. During the Covid19 pandemic, many students had to stay at home and continue their education online and experienced changes in their emotions [14,15]. Al-Obaydi et al. [16], investigated cognitive, behavioural, and emotional engagement in online classes and stated that increased student engagement is not always due to the use of online platforms, and that it is very important to carefully plan educational tactics to increase and maintain student engagement. While negative emotions decrease motivation [17], positive emotions are beneficial in generating innovative ideas and strategies [18]. Teachers’ emotions are of importance in their efficiency [19,20]. As a result of the analysis of studies on teachers’ emotions conducted between 1985 and 2014, Uitto, Jokikokko, and Estola [21] emphasized the importance of teachers’ emotions.

When the relevant literature is examined, seven different studies conducted in different geographical regions and languages, studies examining teachers’ work stress, burnout, self-efficacy, work commitment and emotional states draw attention. Ghasemzadeh, Nemati and Fatti [22] investigated the burnout-related variables of Iranian EFL teachers and showed that teacher reflection and self-efficacy were effective in predicting burnout. Data obtained from 171 participants were evaluated by SEM. They showed that teacher reflection on burnout explained 12.1% of the variance and 25.2% of teacher self-efficacy. Although both variables had a unique effect on teaching burnout, teacher self-efficacy appeared to be a stronger predictor of burnout. Carroll et al.[23], in a survey of 749 teachers in Australia, stated that more than half of the teachers were stressed and those at the beginning of their careers thought of leaving the profession, while primary school teachers and teachers working in rural and remote areas were at the highest levels of stress and burnout. Sokal, Trudel and Babb [24] examined the burnout status of Canadian teachers in the Covid-19 process with 1278 participants. As a result of the study, he emphasized that the "initial exhaustion" variable is an important indicator. Han and Wang [25] evaluated the relationships between self-efficacy, work engagement and reflection in a positive context of English teachers in China and emphasized the importance of emotional state in online lessons. A survey was conducted with the experimental group consisting of 614 participants with various experiences and academic degrees, and as a result of the study, it was emphasized that more time and energy could be spent on supporting pedagogical subjects as well as psychological factors in teaching the English language. Fan and Wang [26] investigated the role of well-being and emotion regulation in the professional success of English teachers in China and revealed that these factors are important variables that affect professional success. The results of the survey conducted with 357 participants were evaluated with the Structural Equation Model (SEM). Structural model results showed a strong, positive link between well-being, emotion regulation, and professional success. The model also showed that both well-being and emotion regulation were highly effective in the professional success of Chinese EFL teachers. Wang, Derakhshan and Azari [27], in their study with 1135 EFL teachers from four Asian countries, found that teachers’ psychological well-being and work engagement positively affect Teacher immunity, which is defined as ’a robust armoring system that emerges in response to high-intensity threats’ by Hiver [28]. Wang [29] examined the emotional state of Chinese English teachers in online classes during the Covid-19 pandemic. Using open-ended questionnaires and semi-structured interviews with 216 Chinese EFL teachers, he found that most teachers found online lessons more boring than face-to-face lessons. All these studies provide important information to understand the effects on teachers’ work stress, burnout and psychological well-being and to support the needs of teachers in education systems. One of the strongest and most natural ways of communicating emotions is facial expressions. It is known that various systems have been developed to detect facial expressions [30]. Among them, the Facial Action Coding System (FACS), developed by Ekman and Friesen in 1978, is referred to as the most widely used as well as the most comprehensive and psychometrically sensitive method [31]. There are a many studies in Facial Action Coding System such as social robots [32,33], medical treatment [34], driver fatigue monitoring [35,36], psychological monitoring [37–39], facial nerve grading in medicine [40], facial image compression and synthetic facial animation [41], video indexing, robotics, and virtual reality [42]. In addition, there are other studies such as examining change in emotional states of university students while interacting with smart learning systems [43], determining facial features to gauage emotional states during interaction in smart learning system [44], use Microsoft cloud-based Facial Emotion Recognizer software [45,46] and researching effectiveness of this software [47], analysis of facial expressions in formal and distance education environment [48–50], application of smart teaching assessment model to determine students’ emotions with facial analysis [51].

If we take a closer look at the educational applications of facial expression reading, Savchenko, Savchenko and Makarov [52] developed and trained a neural network for face detection in his study in which he analyzed the behavior of students in the e-learning environment. After fine-tuning the software, facial features have been extracted from the detected face image and it was shown that the resulting facial features could be used for rapid simultaneous estimation of students’ participation levels, individual emotions, and group-level emotions. A concentration index was created for students in a study [53] in which the data obtained from pupil tracking and head movements, as well as the emotions of students’ participation level, were combined. It was determined that the students who got the best scores had higher concentration indexes.

Gupta, Kumar and Tekchandani [54] calculated the engagement index (EI) to determine whether the students were related to the course and according to this value, the student was classified as “Engaged” or “Disengaged”. As a result of the studies carried out by training different deep learning models (Inception-V3, VGG19 and ResNet-50) on different datasets (FER-2013, CK+ and RAF-DB), the system was tested with 20 students and the ResNet-50 model showed 92% accuracy in classifying facial emotions. It was determined that it performed better than other models. Ma et al.[55] proposes and implements a real-time emotion score identification model based on convolutional neural network. It captures an image of students during training via a webcam, evaluates students’ learning feelings from this image in real time, and provides real-time feedback to the lecturer in distance learning. The FER2013 dataset was used to train the CNN model to be used to extract facial expressions. As a result of the study, it was found that the instructors who used the online emotion recognition model were able to obtain the emotional state of the students more intuitively than the instructors who did not use the emotion analysis model. Based on this feedback, instructors make time adjustments to the lessons, add analysis of examples, or communicate with students by asking questions. Using the emotion-recognition model, students can communicate more with their instructors and get support quickly when they have problems. Some lecturers also refused to use the system, "thinking that the lecturer interfered with the idea of teaching". It is seen that a similar study (with the same software architecture and dataset) was done by Divya et al [56]. Pabba and Kumar [57] conducted a similar study with BAUM-1, DAiSEE and YawDD datasets and reached training and testing accuracy of 78.70% and 76.90%, respectively. This accuracy rate was found to be 93.63% by Sun et al.[58]. In a study in which a smart application was developed for teachers, which understands the emotions of the students and determines the participation levels of the students during the lesson, the face region was detected with the OpenCV software library, then 7 different emotion detection and eye opening detection were made using CNN from the face image. Concentration calculations were made by combining these data. The concentration index is used to classify the student as "Highly-engaged", "Engaged", and "Disengaged". The developed application was tested on a 28-second lecture video from YouTube consisting of 11 students [59]. Hou et al.[60] used automatic face recognition and emotion recognition to solve the problem of teachers’ inability to understand students’ listening status, which they stated as one of the disadvantages of online teaching. Student concentration level was tried to be extracted from 7 different emotion data obtained. It has been stated that the software using software modules such as OpenCV, MTCNN and VGG16 has application value in related fields. In a study conducted to determine the learning effect of students and the changes in the teaching quality of teachers due to the lack of emotion arising from the time and space differences between students and teachers in distance education, a student’s emotion recognition model is proposed in an online learning environment and an intelligent education system called Smart-E is established. This model uses OpenCV and DLib software libraries for face recognition, deep neural network and support vector machine for emotion recognition. The facial images of the students were defined in real time and the direction and size of the emotional vector of the students were calculated from the expression classification and blink frequency to understand the emotional states of the students [61]. Wang et al. [62] reported a lack of direct, timely and effective communication and feedback between teachers and students in online courses compared to traditional face-to-face classes. In this direction, in their study, they proposed a framework that combines a facial expression recognition (FER) algorithm with online course platforms in terms of computer simulation. The cameras in the devices collect the facial images of the students and their facial expressions are analyzed and classified as 8 types of emotions with the FER algorithm. An online course with 27 students conducted on Tencent Meeting was used to test the proposed method, and the result proved that this method performs well in different environments. Yang et al. [63] in his study, an input to the neural network was created by performing feature extraction from face images, subset feature extraction and emotion recognition, and emotion estimation was obtained from the face photo as a result of the model. As a result of the tests conducted using The Japanese Female Facial Expression (JAFFE) Dataset, they stated that emotion recognition based on facial expressions is applicable in distance education. The facial expression detection process is used not only for online lessons, but also for creating adaptive learning content in MOOC content. In the study of Soltani, Zarzour and Babahenini [64], students’ facial emotion data were instantly extracted and evaluated by the system.Considering the above-mentioned issues, it is seen that the studies conducted are generally student-centered, and the number of studies with instructors, which is an important element of education, is scarce. In the literature review, no study was found to determine the emotions of teachers, especially teachers who teach through distance education, using the face analysis method. It has become a necessity to investigate how educators who had to continue to teach at home instead of school, as before the pandemic, were affected by this situation. This study aimed to examine the emotional states of the instructors from their facial expressions while they were teaching in the school environment (e-studio) and at home. For this purpose, in this study, it was investigated how the emotions of the instructors changed during the distance education courses in the e-studio and home environment and whether there was a significant difference according to these two environments. Although there are many studies on burnout in teachers and even burnout during the pandemic in the relevant literature, this study was conducted on distance education courses, with Turkish teachers, and with a non-destructive method (that is, objectively measuring the participant’s condition with high accuracy). Therefore, it can be said that this study is important in terms of increasing the quality of original and distance education.

## Materials and methods

In this study, the emotional states of teachers who teach via distance education and the effect of the environment in which they teach on their emotions were examined. The computer aided "Facial Expression Coding System" method was used to measure the emotional values from facial expressions. Through the software developed by the researchers, 43292 facial images taken from five teachers were analyzed during their training and seven basic emotions representing facial expressions were numerically obtained.

When the study started, there were Covid-19 Pandemic conditions and a curfew was applied in our country. During the curfew, the vast majority of government institutions were suspended and the boards became inoperative. For this reason, ethical approval could not be obtained from the ethics committee for the study, but verbal consent was obtained from the participants before starting the study. Participants who did not want to participate were not included in the study. Upon the end of the curfew, the consent form was signed by the participants. Consent forms have been uploaded to the system.

### Study group

This study was conducted at a state university in the Mediterranean Region with a total of five instructors (3 men and 2 women). The ages of the instructors participating in the study ranged between 36 and 38, while their teaching experiences in distance education ranged from 8 to 10 years. Necessary permissions to analyze the lecture videos recorded during the semester were obtained from the instructors at the end of the semester, which prevented any effect on their emotional states during the course. The summary table containing the information of the participants is given below (Table 1).

**Table 1:** Summary information of the participants.

ID	Gender	Ages	Distance Education Experiences (Year)
Instructor 1 Instructor 2 Instructor 3	Man Man Man	38 36 36	9 8 8
Instructor 4 Instructor 5	Women Women	38 38	10 8

### Facial emotion recognition

Facial Action Coding System (FACS) was used in the study to determine the changes in emotions of instructors. In the early twentieth century, Ekman and Friesen [65] identified seven basic emotions based on a cross-cultural study, which showed that people reflect some basic emotions in same way regardless of culture.

**Fig 1:**
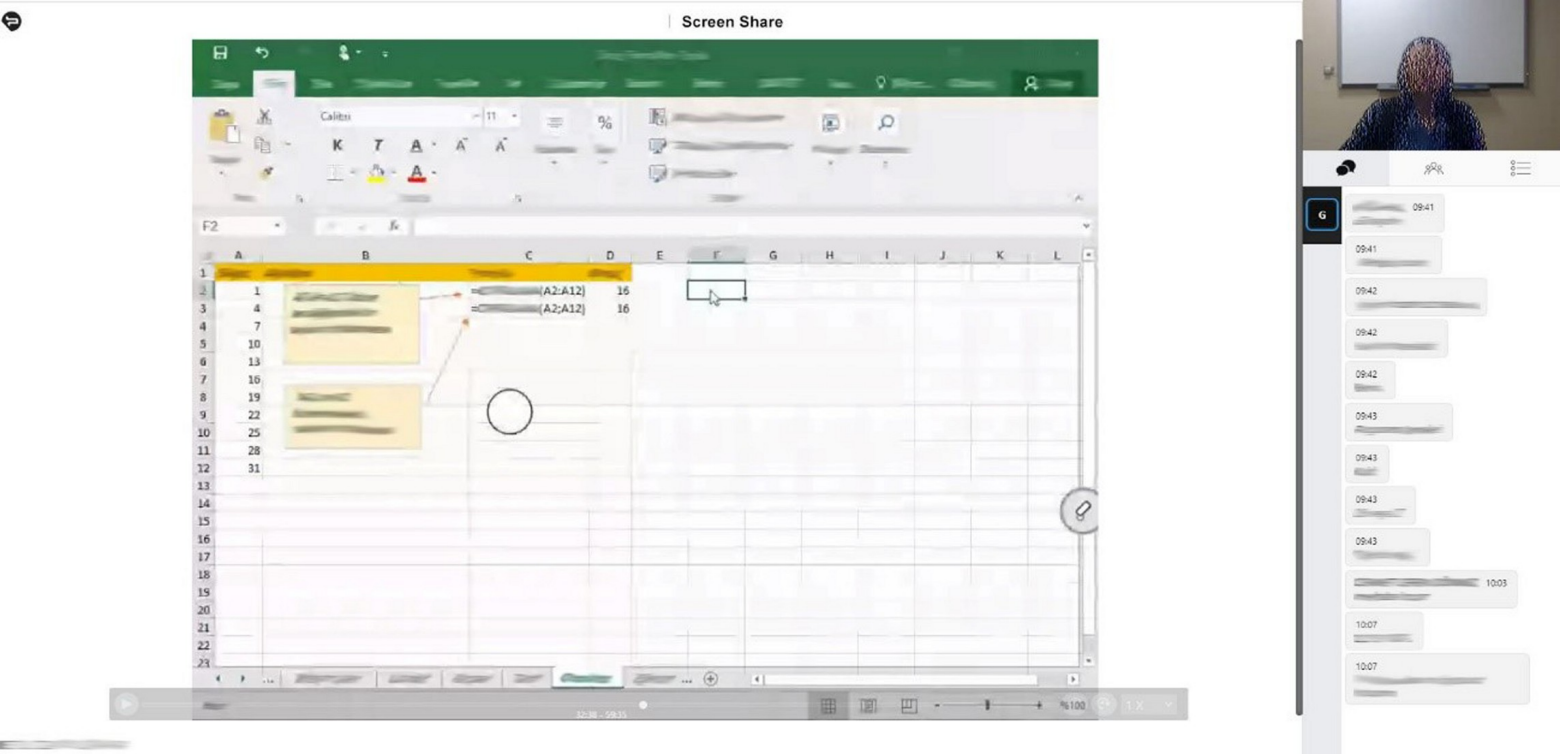
Screenshot of training videos in which the images of the trainers are cropped.

**Fig 2:**
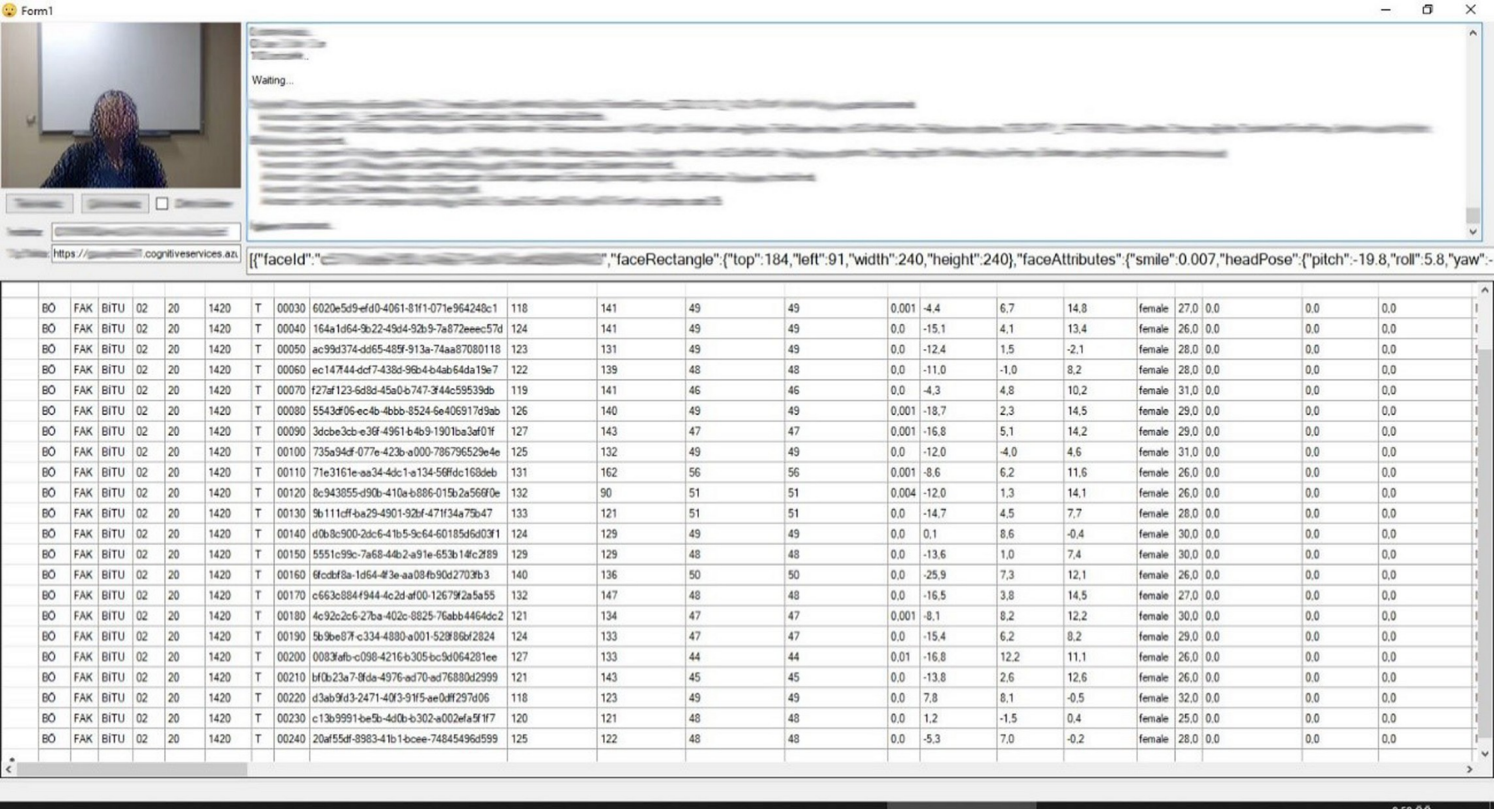
Screenshot of the software with which the photos were analysed.

Computer software can determine facial expressions with great accuracy with the help of image processing algorithms to detect certain points on the face of the person and artificial intelligence algorithms trained with various datasets [66–68]. The feelings of disgust, sadness, happiness, fear, contempt, anger, and surprise, which are considered to be universal, were analyzed using a software developed by the researchers via the C# programming language and the Microsoft Face Recognition API in the Visual Studio software development environment from instructors photos. Photos of the instructors involved in the study were cropped from the recorded course videos (Fig 1) at 10-second intervals and analyzed with the specified program (Fig 2). The photographs in which the faces of the instructors could not be seen completely due to various reasons were deleted and excluded from the study. The study was carried out using a total of 43,292 photos in which the instructors’ faces can be fully seen and the analysis was performed successfully. The photos contain images of the lecturers in their e-studio and home environments. Fig 2 shows the screenshot of the software developed by the researchers in which the photographs were analyzed.

### Data analysis

With the computerized facial movements coding system, the changes in the emotions of the instructors were digitized and the changes were examined by visualizing them using a pivot chart. The change in each emotion is shown in the graph as a series. The pivot function calculates the arithmetic average of the relevant emotion values of all instructors in that second. This process was repeated for the series representing each emotion [69].

In order to determine whether the changes were significant or not, the normal distribution of the data was examined and the data that was not normally distributed were tried to be normalized [70–72], but it was determined that the data did not follow normal distribution. Therefore, Mann Whitney-U test, one of the non-parametric tests, was used to determine whether the difference was significant [73,74].

## Results

**Fig 3:**
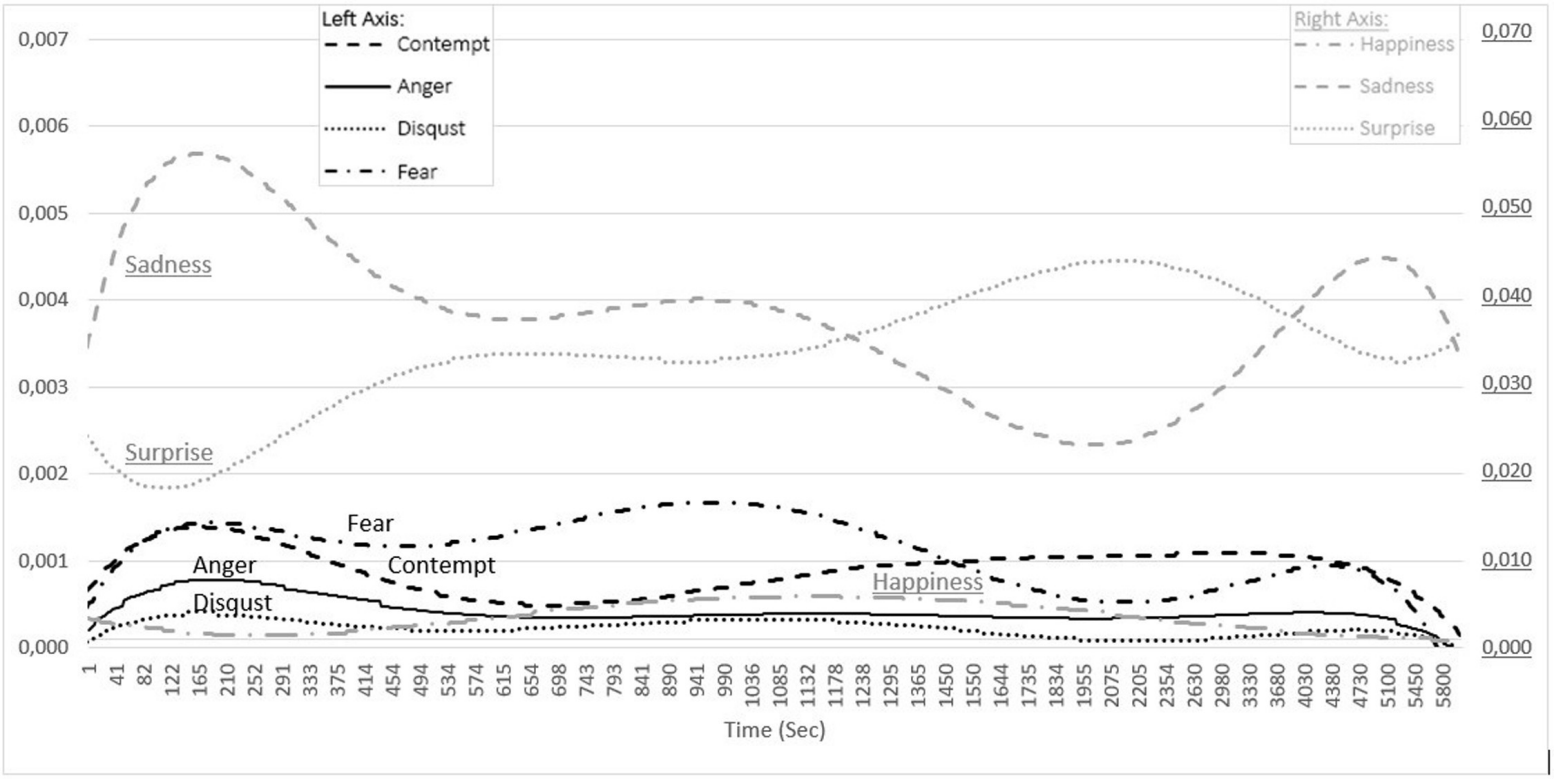
The change in the emotions of the instructors during the lectures held in the e-studio.

**Fig 4:**
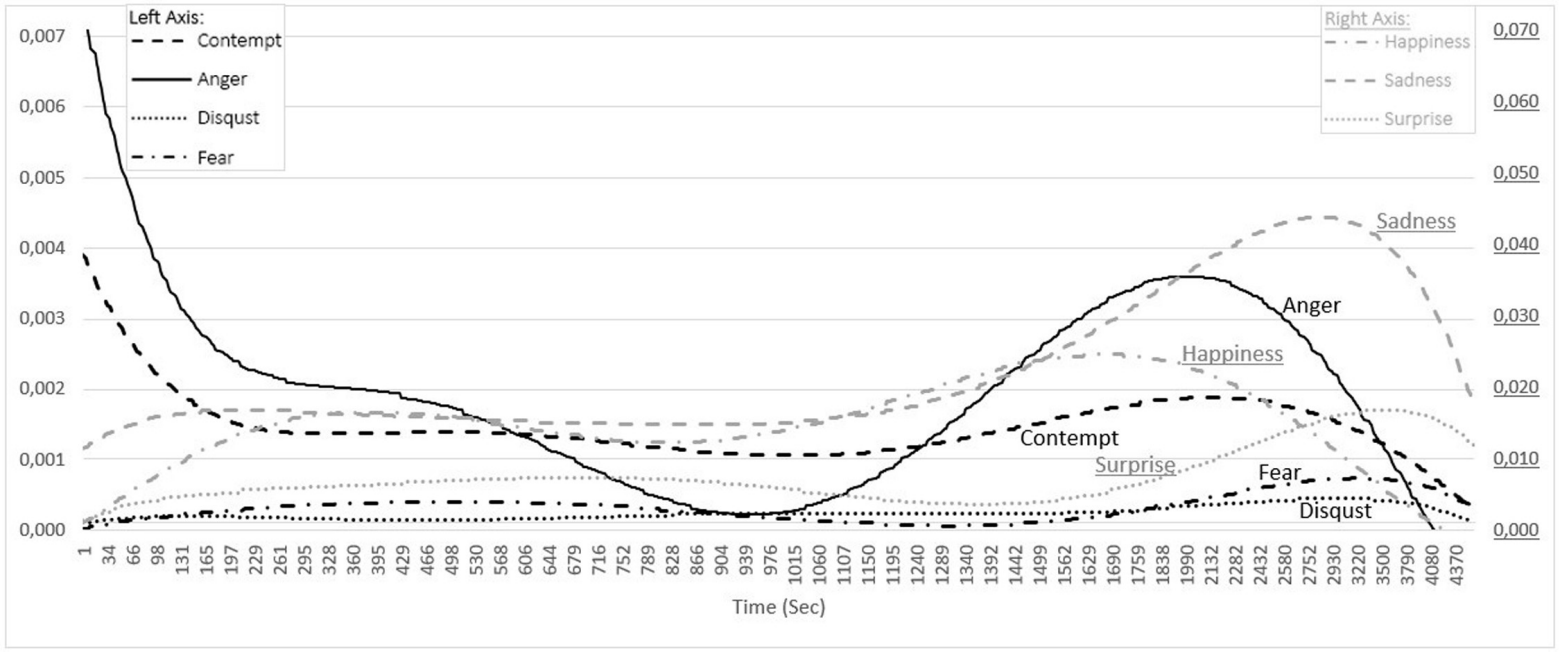
The change in the emotions of the instructors during the lectures held at home.

This study examined how the emotions of the instructors changed during a distance education course. The emotional changes of the trainers in the e-studio environment and in the home environment were expressed with graphics (Figs 3 and 4). In the spring semester of the 2020–2021 academic year, the lectures were conducted lessons from the e-studio in the first 7 weeks and from the home environment during the next 7 weeks. As a result of the FACS analysis, the total value of the instructors’ feelings of sadness, surprise, fear, contempt, anger, disgust, and happiness during the lectures is equal to 1. The feeling of neutral is not included in the chart because it reflects neither positive nor negative emotions. In the graph, each series represents a change in a specific emotion. While calculating the values that make up these series, the arithmetic average of the related emotional values of all the instructors in that second was taken. This process was repeated for the serie representing each emotion. As seen in the graph, the second values range from 1 to 5800. Contempt, Anger, Disgust and Fear are on the left axis, while Happiness, Sadness and Surprise are on the right axis.

Fig 3 shows the changes in the emotions that was determined as a result of the analysis of the videos of the lectures conducted by the instructors in the e-studio environment in the first seven weeks of the term. According to this graph, it is clear that, while Happiness and Surprise declined at the beginning of the lecture, other emotions increased. The graph reveals that each emotion saw a different fluctuation according to the time. Sadness started to rise at the beginning of the lecture and reached a high between approximately the 1st minute (41st sec) and the 4th minute (252th sec). After this period, it decreased until about the 10th minute (574th sec), followed by a slight rise, and then started to drop rapidly after approximately the 17th minute (1036th sec). Sadness, which sharply rose again after approximately the 35th minute (2075th sec), declined after approximately the 85th minute (5100th sec). On the other hand, the change in Surprise was almost the opposite of the change in Sadness. Surprise, which decreased at the beginning of the lecture, started to increase rapidly after about the 2nd minute (122nd sec) until about the 9th minute (534th sec). Afterwards, there was no sharp change despite the rise until the 17th minute (1036th sec). However, it went up rapidly again from about the 17th minute until about the 35th minute (2075th sec), and then started to diminish after about the 85th minute (5100th sec). Fear, Anger, Contempt, and Disgust saw a rise from the beginning of the lecture until approximately the 4th minute (252th sec), and then started to decrease. Fear diminished from approximately the 4th minute (252nd sec) until approximately the 9th minute (534th sec), after which it increased until approximately the 17th minute (1036th sec). It declined until about the 35th minute (2075th sec) after the 17th minute, and then rose until approximately the 73rd minute (4380th sec), following which it started to drop again. Contempt decreased from approximately the 4th minute until approximately the 12th minute (743rd sec), and then increased until approximately the 73rd minute (4380th sec) and declined rapidly after this period.

On the other hand, after Anger and Disgust declined from approximately the 4th minute (252nd sec) until approximately the 9th minute (534th sec), they saw only slight changes. Finally, after Happiness started to decrease from the beginning of the lecture until about the 4th minute (252nd sec), it increased until about the 21st minute (1295th sec). It, then, dropped until the end of the lecture.

The study revealed that, during the lectures taking place in the home environment, the emotions were different from those in the e-studio environment (Fig 4). It was determined that while Anger and Contempt were quite high at the beginning of the lecture, they fell sharply until about the 4th minute (229th sec). Although it was much more evident in Anger, both emotions continued to decrease until about the 16th minute (939th sec). Contempt tended to decrease very minimally between the 4th and 16th minutes, though the change was pretty vague. Both emotions increased after about the 16th minute until about the 32nd minute (1990th sec) and then declined rapidly until the lecture was over. The graph shows that Sadness increased in the first 4 minutes of the lecture, yet its value was not as high as in the school environment. Although there was a slight decrease in Sadness from the 4th minute (229th sec) until approximately the 19th minute (1107th sec), there was no significant change. However, afterwards, it increased rapidly until approximately the 49th minute (2930th sec) and then decreased sharply. There were no significant changes in Fear and Disgust. Yet, they saw a slight increase after about the 28th minute (1690th sec) and a decrease after about the 49th minute (2930th sec). Surprise started to increase slightly at the beginning of the lecture and continued to rise until approximately the 13th minute (789th sec). After that, it decreased until the 22nd minute (1340th sec), increased until the 58th minute (3500th), and then started to decline again. There was a slight decline between the 9th minute (568th sec) and 14th minute (866th sec) in Happiness, which rose rapidly at the start of the lecture, although there was no significant change. After that time, happiness saw an increase until the 28th minute (1690th sec) and then dropped until the end of the lecture.

The instructors, who had taught in the e-studios at the university in the first 7 weeks of the study carried out in the 2019–2020 Spring semester, were obliged to teach at their homes in the following 7 weeks due to the pandemic. Mann Whitney-u test was used to analyze whether the environment significantly differentiates the emotions of the instructors (Table 2).

**Table 2:** The change in the emotions according to the environment where instructors taught Mann Whitney-u test.

Emotion	Environment	Mean Rank	Sum of Ranks	U	p
Anger	e-studio	206.13	64520.00	15379.000	.072
	home	230.45	25580.00		
Contempt	e-studio	222.60	69672.50	14211.500	.004
	home	184.03	20427.50		
Disgust	e-studio	219.94	68841.50	15042.500	.034
	home	191.52	21258.50		
Fear	e-studio	229.84	71941.00	11943.000	.000
	home	163.59	18159.00		
Happines	e-studio	203.92	63826.50	14685.500	.015
	home	236.70	26273.50		
Sadness	e-studio	235.35	73664.50	10219.500	.000
	home	148.07	16435.50		
Surprise	e-studio	230.37	72105.00	11779.000	.000
	home	162.12	17995.00		

The analysis concluded that all the emotions except Anger changed depending on the environment. It is clear that the significant difference in Contempt (U = 14211.5, p < .05), Disgust (U = 15042.5, p < .05), Fear (U = 11943.0, p < .05), Sadness (U = 10219.5, p < .05), and Surprise (U = 11779.5, p < .05) was higher in the e-studio environment. On the contrary, it was determined that Happiness was higher in the home environment (U = 14685.5, p < .05).

## Discussion

Stressful situations or emotional fatigue can distract teachers and make it difficult for them to provide effective teaching, with negative consequences such as student apathy, low motivation and behavioural problems.

As a result, teachers’ emotional state significantly affects their education and learning processes. Positive emotional states have a positive effect on student achievement and classroom climate. Therefore, paying attention to and supporting teachers’ emotional needs will contribute to the creation of healthier and more productive teaching and learning environments.

The study revealed that, in the lectures held in the e-studio environment, Sadness felt by the instructors increased at the beginning of the lecture and reached a high in about the 3rd minute (165th sec) and then a low in about the 35th minute (2075th sec). It was determined that Sadness saw a progressive change in the form of continuous fluctuations. The fall in Sadness, which started after the 3rd minute continued until about the 10th minute (574th sec), and then saw a slight rise until approximately the 17th minute (1036th sec). The study discovered that, a negative emotion, Sadness, which saw its first decline after the introduction of the lecture, started to decrease again after the 17th minute, which was close to the time when some instructors had a break. This can be attributed to the decrease in the negative load on the instructors who would have a break [75]. It was observed that Sadness increased again after the 35th minute of the lectures in which the period before the breaktime lasted longer, and that there was a decrease in it in the last part of the lectures. This can be considered as the impact of the duration on the emotion [76].

Surprise tended to be just the opposite of Sadness. Surprise reached a low in about the 2nd minute (122nd sec) and a high in about the 35th minute (2075th sec). The continuous increase (both slight and significant) in Surprise from the second minute until the 35th minute of the lecture can be considered as a positive aspect experienced by the instructor. Similarly, Boonroungrut, Oo and One [47] interpreted the feeling of surprise as a positive emotion according to the Russell’s circumflex model. This situation can also be interpreted as an increase in teachers’ self-efficacy beliefs as the course duration progresses [77].

Fear, Anger, Contempt, and Disgust (negative emotions) increased at the start of the lecture until approximately the 4th minute, and saw fluctuations afterwards. The change in Fear and Disgust occurred at almost the same period of time and in the same direction with Sadness. Anger, also, saw a similar change to these negative emotions. The feeling of fear occurs as a result of a threat felt in the environment, and it results in escaping from the environment, struggling with the problem, or being unable to do anything [78]. Fear observed in the lecturers’ emotions indicates that an anxiety occurred in the first minutes of the lectures, after which it disappeared. A similar change was experienced with the feeling of surprise at similar times and was associated with the instructor’s self-efficacy [77]. Fear rose again as the lecture progressed, yet there was a significant decrease in most of the lectures when approaching the breaktime.

Contempt declined from the 3rd minute until the 12th minute of the lecture and then saw an increase until the 73rd minute, following which it fell rapidly. One can argue that Contempt, a negative emotion, generally tended to increase. On the other hand, Anger and Disgust increased in the first minutes of the lecture and afterwards dropped until the 9th minute. After that time, there was not any significant change in both of them. Izard [79,80] states that the feelings of contempt, anger, and disgust are generally experienced together by individuals. Similarly, this study showed that the directions of change in Anger and Contempt and their durations were close to each other, and yet Disgust differed in some periods of time [81]. It can also be stated that the feelings of anger and disgust were not experienced very intensely.

Happiness, which is a positive emotion, decreased at the start of the lecture and increased after about the 4th minute until about the 17th minute. After about the 21st minute, there was a decrease in it until the lecture ended. The graph shows that negative emotions increased in the first minutes of the lecture, but this changed rapidly and positive emotions increased when the lecture started actively. Another decrease in happiness observed after the 21st minute suggests that the duration has an effect on happiness along with fatigue [82]. In addition, it is clear that the value of Happiness is not very high. Similarly, some studies revealed that teachers experienced negative emotions more than positive emotions [83,84]. One can suggest that the pandemic experienced at the time of the study might have had deteriorated this.

As far as Anger and Contempt are concerned, they had very high values at the beginning of the lecture conducted in the home environment and decreased quite sharply until about the 4th minute. The fall in these two emotions continued until about the 16th minute, more sharply in Anger and slightly in Contempt. After this time, there was an increase until the 32nd minute and then a decrease until the end of the lecture. That Anger had such a high value at the beginning of the lecture may be attributed to the fact that the feeling of anger is related to the perception of injustice [85] and the feeling of frustration [86]. It can be argued that, while negative emotions of the individuals caused by the Covid-19 pandemic may have triggered this emotion, the lectures created positive reflections on the emotions of the instructors [27,29]. Although there was a slight rise in Sadness at the beginning of the lecture, it did not see a serious change for a long time after about the 3rd minute. However, after about the 17th minute, it increased rapidly until about the 49th minute, and after this time it declined swiftly. As for Fear, at first there was a slight decrease and then a minimal increase. The most significant change in this emotion was observed as a rise between the 28th and 54th minutes and then a fall until the end of the lecture. In general, the study showed that the changes in negative emotions were in the pattern of an initial decrease, followed by an increase and then another decline. It is clear that Fear and Disgust did not have very high values. Similarly, Hosotani and Matsumura [87] stated in their study that teachers experience feelings of fear and disgust less than other emotions.

The study revealed that, a positive emotion, happiness continued to increase for a long time. This change in emotions suggests that, although the instructors experienced negative feelings such as anger and contempt intensely in the home environment during the pandemic period, they rapidly decreased, while happiness, which is a positive feeling, increased at the start of the lecture due to the feeling of safety provided by the home environment. In addition, this change may have resulted from the dissolution of the stress caused by the pandemic period and the long duration spent at home. The study revealed that the fluctuations in the emotions during the lecture occurred in accordance with the natural progression of the lecture.

It was determined that there was a significant difference in the other six emotions of the instructors, except for Anger, depending on the environment in which the lecture took place. It was discovered that, negative emotions, Contempt, Disgust, Fear, and Sadness were higher in the e-studio, while Happiness, a positive emotion, was higher in the home environment. Surprise was also observed more in the e-studio. Another study found out that, while teachers generally feel positive about their students, they experience negative emotions about difficult students their parents and colleagues [88]. One can say that different environments cause changes in emotions. Although it was not included in the study, it can be argued, as a result of the interviews with the instructors, that there may be two reasons for the differentiation in the values. The first is the effect of the physical conditions in the studio. Despite the homogeneous and clear sound in the studio environment [89], physical conditions such as too small environment and lack of ventilation may increase negative emotions. Secondly, the feeling of safety created by the instructors’ staying at home during the pandemic period may have reduced the negative emotions, or the individuals who were constantly at home might have desired to evaluate the time they spent during the lecture positively.

### Limitations and further studies

One of the conclusions that can be drawn from this study is that it is recommended that distance education instructors should teach without depending on any location. This study was carried out on certain courses with the participants given under the title of "Study Group". It would be beneficial to conduct similar studies in different types of course groups and education levels. From the point of view of educators, it can be suggested that instructors take a break when they feel that negative emotions increase during the lesson. It is thought that more studies can be done on the emotions of the instructors and students during the lessons, which are the basic components of distance education. Since emotion is a complex structure and different emotions may differ between cultures, it would be beneficial to repeat such studies for different cultures and use their outputs to increase the quality of distance education.

## Supporting information

S1 Graphical abstract (DOCX)
