# Supplementary material for: Effect of distance education courses held in different environments on emotions of the instructor
Source: PLoS One. 2024 Jan 26;19(1):e0295935. doi: 10.1371/journal.pone.0295935 (PMC10817226; doi:10.1371/journal.pone.0295935)
Supplement: S1 Graphical abstract — (DOCX) [file pone.0295935.s001.docx]

**Graphical Abstract**

Effect of Distance Education Courses Held in Different Environments on Emotions of the Instructor

**Note for design:**

Dear editor,

I designed this graphical abstract so that the work can be better understood and attracted by the readers. I purchased the background image in the content of the visual from the istock website. I am willingly ready to make any necessary adjustments to the visual.

I upload the image in docx format to the system, if you wish, I can also forward it to you in Photoshop (.psd), Acrobat (.psd) or JPEG (.jpg) Image format.

Best regards.
